# Case Report: A case of *COL1A1*–*PDGFB* fusion uterine sarcoma at cervix and insights into the clinical management of rare uterine sarcoma

**DOI:** 10.3389/fonc.2023.1108586

**Published:** 2023-03-13

**Authors:** Linghui Lu, Shunni Wang, Haoran Shen, Feiran Zhang, Fenghua Ma, Yue Shi, Yan Ning

**Affiliations:** ^1^ Department of Pathology, Obstetrics and Gynecology Hospital of Fudan University, Shanghai, China; ^2^ Department of Gynecological Oncology, Obstetrics and Gynecology Hospital of Fudan University, Shanghai, China; ^3^ Department of Radiology, Obstetrics and Gynecology Hospital of Fudan University, Shanghai, China

**Keywords:** COL1A1-PDGFB fusion, uterine sarcoma, RNA sequencing, dermatofibrosarcoma protuberans (DFSP), NTRK fusion, target therapy

## Abstract

*COL1A1*–*PDGFB* gene fusion uterine sarcoma is an especially rare malignant mesenchymal tumor that was previously classified as an undifferentiated uterine sarcoma due to the lack of specific features of differentiation. Till now, only five cases have been reported, and here we presented another case recently diagnosed in a Chinese woman who had vaginal bleeding. She presented with a cervical mass at the anterior lip of the cervix invading the vagina and was treated with laparoscopic total hysterectomy plus bilateral salpingo-oophorectomy (TH+BSO) and partial vaginal wall resection with the final pathology of *COL1A1*–*PDGFB* fusion uterine sarcoma. Our aim is to emphasize the importance of differential diagnosis of this rare tumor, as early precise diagnosis may allow patients to benefit from the targeted therapy imatinib. This article also serves as further clinical evidence of this disease, serving to increase clinical awareness of this rare sarcoma to avoid misdiagnosis.

## Introduction

Uterine mesenchymal tumors consist of a group of heterogeneous tumors with various morphological features, immunohistochemical (IHC) presentations, and genetic mutations. Undifferentiated uterine sarcomas are malignant mesenchymal tumors that lack specific features of differentiation and are diagnosed with the exclusion of others. With the rapid development of molecular technology including fluorescence *in situ* hybridization (FISH) and next-generation sequencing (NGS), the pathological classification and prognosis assessment of these tumors achieved remarkable progression (1, 2).

*COL1A1* gene is located at chromosomes 17q21.3 to q22.1 with 52 exons. The gene is highly variable, as it contains multiple fragile breakpoints spanning a wide range. *PDGFB* gene is located at chromosomes 22q12.3 to q13.1 containing 7 exons. Its breakpoint is consistently present in intron 1. When these two genes merged, the expression of *PDGFB* would be muted from the regulation of upstream inhibitory factors, and *COL1A1*–*PDGFB* chimeric mRNAs would be generated, resulting in *PDGFB* and its receptor (*PDGFBRB*) stimulating cell proliferation in an autocrine or paracrine manner, which was reported to be oncogenic (3–5).

According to the previous literature, *COL1A1*–*PDGFB* gene fusion occurred mainly in soft tissue dermatofibrosarcoma protuberans (6–8) and pediatric giant cell fibroblastoma (9), while it was rarely reported in the female genital tract. Croce (10) first reported three cases with *COL1A1*–*PDGFB* fusion in uterine sarcomas in 2019, and subsequently, Samuel and Adriana respectively reported one case each in 2020 and 2022 (11, 12).

The relevant literature had been reviewed, and no reports in China had been found on this tumor so far. Here, we presented the first uterine sarcoma located at the cervix with *COL1A1*–*PDGFB* gene fusion in China.

## Case report

### Clinical presentation

A 57-year-old woman presented with uninduced post-menopausal vaginal bleeding for 2 weeks. Gynecological examination revealed a 4-cm mass on the anterior lip of the cervix protruding to the vagina. Pelvic ultrasound showed a 51 × 45 × 35 mm^3^ hypoechoic mass in the lower segment of the uterus extending to the anterior lip of the cervix with a rich blood supply. MRI displayed an irregular exophytic mass on the cervix that presented slightly high signal intensity on T2-weighted imaging (T2WI) with significant enhancement (**Supplementary Figure 1**), and cervical myoma was suspected for which malignancy could not be excluded. As for medical history, she had undergone surgery for papillary thyroid cancer in another hospital with iodine-131 radiation after an operation in 2011, and regular follow-ups showed no abnormality currently. She denied any family history. For obstetric history, she was G2P2 with two children born through vaginal delivery in her 30s. She denied any unprotected sex, and no bleeding was noticed during intercourse. The pre-op tumor markers were all within normal range. A comprehensive pre-op evaluation was performed with the human papillomavirus (HPV) test as negative, liquid based cytology test (LCT) as negative for intraepithelial lesion or malignancy (NILM), and abdominal+chest CT with contrast showing no suspicious lymph node or any other abnormality.

As cervical myoma was considered and malignancy could not be excluded, total hysterectomy plus bilateral salpingo-oophorectomy (TH+BSO) plus partial vaginal wall resection was suggested for this patient who has had menopause for 11 years already. The patient received laparoscopic surgery with frozen pathology reported as cervical myoma, and the whole specimen was extracted through the vagina. As the tumor size was slightly too big to pass through the atrophic vagina, the uterus was dissected under the protection of a specimen bag, and there was no dissemination of the tumor during the operation. The tumor was completely resected, and no extrauterine lesions were detected during the operation. Postoperative RNA sequencing of tumor tissue was performed, which supported the diagnosis of *COL1A1*–*PDGFB* fusion uterine sarcoma. Considering the rarity of this tumor and limited data available as to the treatment and prognosis, thorough communication with the patient was conducted, and the decision was reached as no further adjuvant therapy was given post-operation and close follow-up was required. The patient was suggested to undergo follow-ups every 3–6 months in the first 2 years post-operation, and the frequency could be extended to 6–12 months since the third year after surgery. The patient was recommended to undergo lifelong follow-ups, and the latest follow-up at 6 months after surgery showed no abnormality. The patient had been compliant with regular follow-ups, and no adverse events have been reported so far.

### Methods

The IHC staining was performed on formalin-fixed and paraffin-embedded (FFPE) tissue blocks automatically (Leica Bond Max, Wetzlar, Germany), and then antibodies (**Table 1**) were applied according to the manufacturer’s instructions. RNA extraction, RNA-seq library preparation, sequencing, and analysis were carried out as previously described (13–15). Total RNA was extracted from FFPE tissue blocks, and rRNA was removed to obtain mRNA, which was then processed into short fragments. The interrupted mRNA segments were reverse-transcribed with random primers. After the synthesis of the first strand of cDNA by reverse transcription, the second strand of cDNA was synthesized, which became double-stranded cDNA. cDNA was purified by Beckman AMPPure XP magnetic beads and repaired at the end, and a sequencing joint was added. The target fragments were recovered by purifying magnetic beads and then amplified by PCR. The library constructed was sequenced by Illumina HiSeq2000.

**Table 1:** Details of immunohistochemical results in this case presentation.

Primary antibody	Tumor stain	Clone	Dilution	Manufacturer
CD34	+	QBEND/10	1:200	Changdao
S-100	−	Polyclonal antibody	1:200	DAKO
ER	−	6F11	1:200	Leica
PR	−	PgR636	1:200	DAKO
Desmin	−	D33	1:1,000	DAKO
Caldesmon	−	h-CD	1:200	GT
SMA	−	1A4	1:100	DAKO
CD10	−	56C6	1:1	Leica
TRK	−	EPR17341	1:100	Abcam
P16	+	6H12	1:1,000	Maixin
P53	Wild type	DO-7	1:300	DAKO
Ki-67	30% +	MIB-1	1:150	DAKO
PHH3	25/10 HPF	Polyclonal antibody	1:50	Zhongshan
CCND1	5% +	EP12	1:100	Zhongshan
BCOR	−	C-10	1:50	Santa
ALK	−	5A4	1:200	Leica
MeLan-A	−	A103	1:100	DAKO
HMB-45	−	Polyclonal antibody	1:100	Changdao
NSE	−	BBS/NS/VI-H14	1:100	DAKO
SMARCA4	+	EPNCIR111A	1:200	Abcam
INI1	+	MRQ-27	1:1	Maixin
CD99	−	O 13	1:100	GT

### Pathologic analyses

#### Histopathologic findings

**Figure 1:**
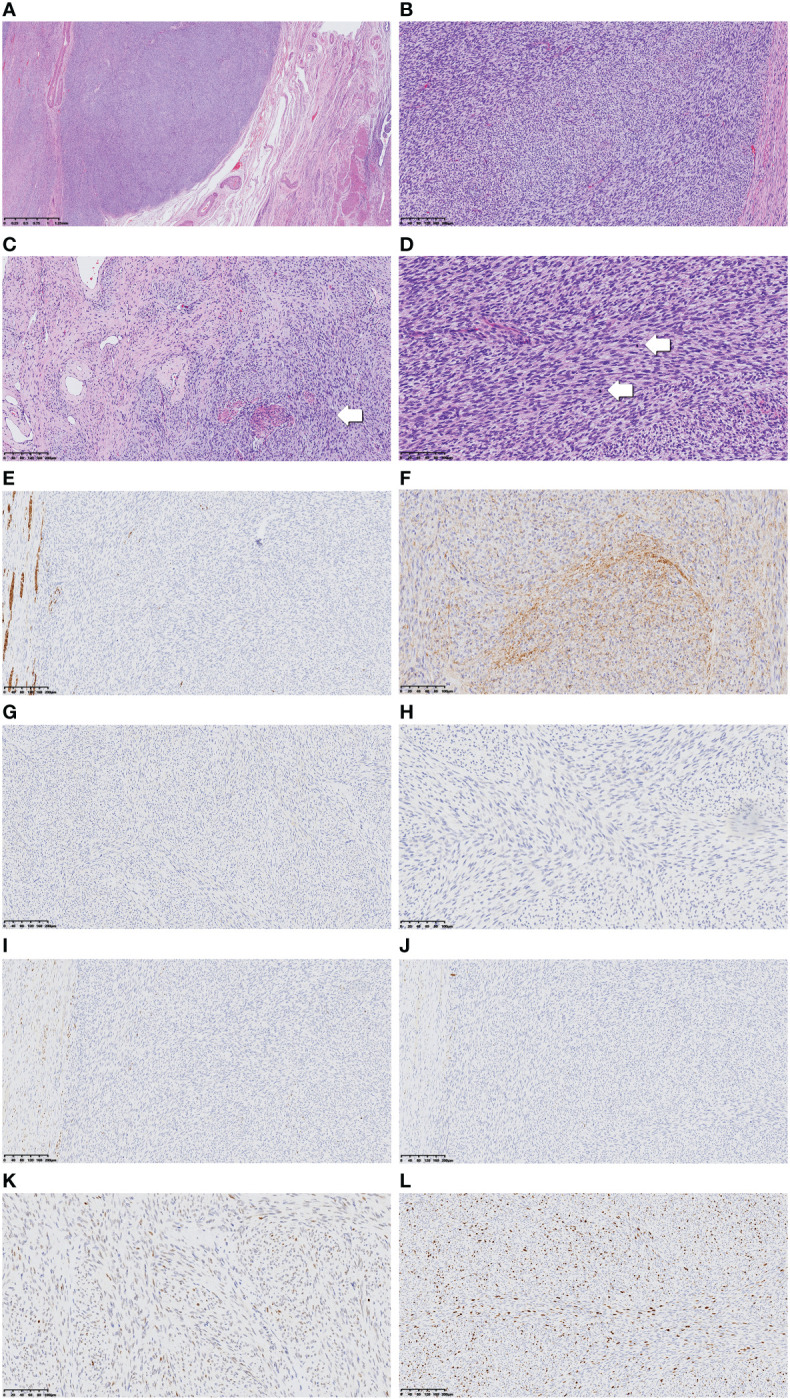
Low-power view of the tumor revealed relatively clear boundary (**A**, ×40). Tumor cells were arranged in a more prominent storiform or herringbone pattern in the cellular view (**B**, ×100), whereas in the cell-sparse area, more dilated small blood vessels can be seen. Normal cervical fibromuscular tissue not invaded by the tumor as pointed by the arrow (**C**, ×100). Tumor nuclei in oval to spindle shape are shown in high-power view, and the cytoplasm appears sparse and eosinophilic. The nuclear heteromorphism appears mild to moderate, while mitoses are numerous and obvious (arrow) (**D**, ×200). Immunohistochemical results including desmin, CD34, TRK, S100, ER, PR, and P53 are displayed (**E–K**, ×200). The Ki-67 proliferation index was high (**L**, ×200).

A gross examination of the specimen revealed a 5.5-cm mass at the anterior lip of the cervix protruding toward the vagina. The tumor cross-section was firm, white, and whorled with relatively clear boundaries (**Supplementary Figure 2**). Microscopically, the tumor boundary was generally clear, while infiltration into the cervical mucosa and fibromyometrium was noticed locally (**Figure 1A**). The tumor consisted of relatively uniform spindle cells densely arranged in more prominent storiform or herringbone patterns. The nuclei were oval- to spindle-shaped, and the cytoplasm was eosinophilic and scarce, with blurred cell boundaries (**Figure 1B**). However, some minor regions with sparse cell distribution and dilated small blood vessels were also seen (**Figure 1C**). Mitoses were relatively active, up to 30 per 10 high-power fields (HPF), with mild-to-moderate nuclear heteromorphism (**Figure 1D**).

#### Immunohistochemical findings

The tumor cells were stained positive for CD34, P16, SMARCA4, and INI1 and scattered weak-positive for CCND1. S-100, estrogen receptor (ER), progesterone receptor (PR), desmin, caldesmon, SMA, CD10, BCOR, ALK, TRK, Melan-A, HMB-45, NSE, and CD99 were stained negative. P53 was stained as wild type, and Ki-67 was expressed in 30% of tumor cells (**Figures 1E–L**).

#### Molecular findings

**Figure 2:**
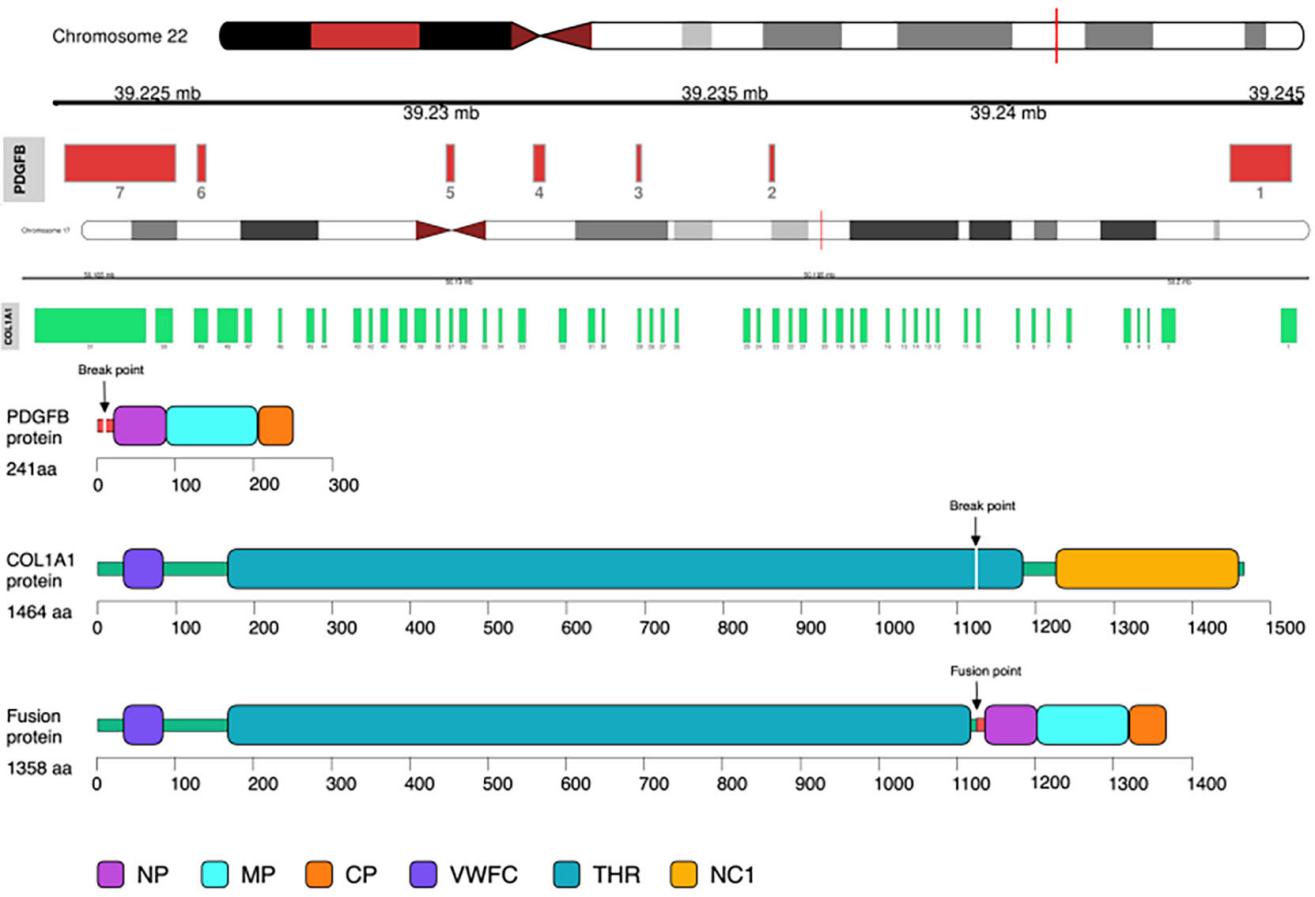
*COL1A1*–*PDGFB* gene fusion was detected by RNA sequencing. Note the unbalanced breakpoints (arrows) on CH17q (*COL1A1*) and 22q (*PDGFB*).

Illumina NextSeq RNA sequencing was adopted, which covered all exons including 632 genes, and special attention was focused on 148 genes (listed in the **Supplementary Material**), and *COL1A1* (NM_000088.3: Exon45)–*PDGFB* (NM_002608.2: Exon2) gene fusion was detected in this case (**Figure 2**).

## Discussion

In 2019, Croce recommended that uterine spindle cell sarcomas could be divided into three categories: *NTRK* fusion group, *COL1A1*–*PDGFB* fusion group, and a group that was tentatively classified as malignant peripheral nerve sheath tumor as positively stained with S100 and contained neither of the molecular abnormalities above (10), a category that excluded leiomyosarcoma (LMS) and high-grade endometrial stromal sarcoma (HGESS). Identification of gene fusion-associated sarcomas is extremely important, as patients can potentially benefit from specific targeted treatments. The first drug that targeted *NTRK* gene fusion-positive tumors and received approval from the Food and Drug Administration (FDA) was larotrectinib in 2018, which had an overall response rate (ORR) of 75% (95% CI: 61–85%, independent review) in a pooled analysis of three phase I and II single-arm trials of 55 combined pediatric and adult patients (16). An updated pooled analysis from three phase I and II clinical trials of larotrectinib (NCT02122913, TNCT02637687, and NCT02576431) resulted in an ORR of 75% (95% CI: 68–81%) based on an investigator review of 206 patients evaluable for response (17). In 2019, entrectinib was also approved, and a pooled analysis of three phase I and II studies showed an ORR of 57% (95% CI: 43–71%, independent review) in 54 adult patients (18). As ORRs for larotrectinib and entrectinib were averaged across different tumor types, the underlying assumption was that the efficacy or effectiveness was the same regardless of histology. As for uterine cancer, the National Comprehensive Cancer Network (NCCN) Clinical Practice Guidelines in Uterine Neoplasms (NCCN Guidelines^®^) explicitly recommended trying either larotrectinib or entrectinib for *NTRK 1/2/3* fusion-positive uterine sarcoma (19). Imatinib was first approved by the FDA for the treatment of patients with soft tissue tumors bearing *COL1A1*–*PDGFB* fusion in 2006 and, according to a recent systematic review, was associated with objective responses in more than 60% of advanced cases (20).

*COL1A1*–*PDGFB* fusion uterine sarcomas were often reported to be asymptomatic. Patient 4 was noticed to have a palpable mass on physical examination, and the tumor grew rapidly during follow-up. The patient in the fifth case came with vaginal bleeding and lower abdominal pain. A cervical mass was found on vaginal examination. The fourth case described that the tumor had a pink–tan–white whorled appearance with areas of necrosis. Our case shared a similar gross appearance, with no obvious necrotic area. Interestingly, our case had a relatively clear boundary and mainly invaded in an expansive manner, compressing on surrounding normal tissue, while local infiltration was seen. Further comparisons between our case and patients reported previously are discussed in detail in **Table 2** (10–12).

**Table 2:** Clinicopathological findings of *COL1A1*–*PDGFB* fusion uterine sarcoma in this case and comparison with previous cases in the literature.

Author and Year	Case Number	Age (years)	Tumor size (cm)	Tumor location	Clinical signs and symptoms	Clinical staging	Follow-up time (months)	Macroscopic appearance	Nuclear atypia	Mitotic figures(per/10HPF)	Tumor Border	Necrosis	Molecular detection method
SabrinaCroce 201910	1 2 3	82 60 48	8.2 5.8 12	Cervical Cervical Corpus	Not available	IB IIIB IB	10ms NED 60ms DOD NA	NA	Mild Moderate Mild	8 20 20	Infiltrating Infiltrating NE	YES NO NO	Array-CGH genomic profile and FISH dual fusion
Samuel L 2020 11	4	43	12	Corpus	Physical examination accidentally found	IVA	34ms DOD	Pink-tan, white whorled	mild	45	Infiltrating	YES	Gene fusion study (RNA sequencing)
Adriana Hogeboom 2022 12	5	50	12	Posterior uterine isthmus extending towards the cervix	Metrorrhagia and lower abdominal pain	IB	2ms NED	Firm, multinodular with white whorled	Moderate	54	Predominant expansile growth	YES	FISH
This study	6	57	5.5	Cervical	Irregular vaginal bleeding	IB	6ms NED	Firm, White whorled	Mild to moderate	30	Infiltrating	NO	Gene fusion study (RNA sequencing)

NED, no evidence of disease; DOD, died of disease; NA, not available; NE, not evaluable; CGH, comparative genomic hybridization; FISH, fluorescence in situ hybridization; HPF, high-power field.

In our case, the uterine sarcoma displayed relatively uniform spindle cells with elongated nuclei, uniform chromatin, sparse cytoplasm, and poorly defined cell boundaries. The nuclei were mildly anomalous, and mitoses were relatively active with up to 30 per 10 HPF, which looked like dermatofibrosarcoma protuberans (DFSP). Previous studies (3, 21, 22) suggested DFSP is a relatively inert, low-to-moderate-malignant soft tissue tumor of the dermis. The morphological feature was poorly defined nodular masses infiltrating subcutaneous or skeletal muscle. The morphology is usually presented with a uniform arrangement of spindle-shaped cell bundles in a typical storiform pattern. Particular attention should be paid to the presence of fibrosarcomatous change or other high-risk features, and CD34 is often highly and diffusely expressed in the cytoplasm. Both histomorphologic manifestations and IHC staining of CD34 overlap with *COL1A1*–*PDGFB* fusion uterine sarcomas. DFSP has a characteristic molecular feature of t(17;22) (q22; q13), and therefore, *COL1A1*–*PDGFB* fusion could be detected in more than 90% of DFSP cases. For DFSP without *COL1A1*–*PDGFB* fusion, the molecular assay showed multiple gene translocations, *P53* mutation or overexpression, or *murine double minute 2* (*MDM2*) overexpression. The most common sites of DFSP were the torso of the body and extremities, and in rare cases, it could also occur at the head and neck, while DFSP in the female genital tract was only reported in the vulvar region. Therefore, although some scholars prefer to refer to uterine tumors bearing *COL1A1*–*PDGFB* fusion as dermatofibrosarcoma in the uterus (considering the morphologic and IHC similarity), the nomenclature of uterine sarcoma with *COL1A1*–*PDGFB* fusion seems more appropriate.

In a pathological setting, leiomyoma, LMS, HGESS, and undifferentiated uterine sarcoma should be considered as the main differential diagnosis of *COL1A1*–*PDGFB* fusion uterine sarcomas, and *NTRK* fusion uterine sarcoma should be the most challenging one to be differentiated (23–25). *NTRK* fusion uterine sarcoma was first reported in 2011 (24, 26) and mainly occurred in young women, with an age range of 23–60 years (average 35 years). The lesions were mostly located at the cervix instead of the corpus, which is the same for *COL1A1*–*PDGFB* fusion uterine sarcomas. The morphology usually presented with fibrosarcoma-like spindle cells arranged in a storiform or fishbone pattern. Other features such as vascular hyalinosis and hemangiopericytoma-like changes, vascular infiltration, and significant inflammatory cell infiltration are rarely seen in *COL1A1*–*PDGFB* fusion uterine sarcomas. IHC markers could be critical clues for diagnosis; usually, TRK, S100, and CD34 were stained positive, with markers of smooth muscle (desmin and caldesmon) and hormone receptors (ER and PR) stained negative for *NTRK* fusion uterine sarcoma. However, the absence or weak expression of TRK cannot rule out the *NTRK* fusion uterine sarcoma due to the poor sensitivity and specificity of IHC staining of TRK, which should be verified by molecular testing if necessary. Of cases of *NTRK* fusion uterine sarcomas, 90% were found to be confined to the uterus at the time of initial clinical evaluation and were potentially responsive to anticancer therapy (27). Boyle and Rabban reported four cases of uterine sarcoma with *NTRK* fusion presented as rare cervical polypoid masses, which could be easily confused with adenosarcoma with stromal overgrowth. However, adenosarcoma usually presented with negative S100 and TRK on IHC, and molecular detection without *NTRK* rearrangement should be the gold standard to facilitate differentiation (28, 29). Similar to *COL1A1*–*PDGFB* fusion uterine sarcomas, both were more common in the cervix and shared similar morphological manifestations. However, the age onset of *COL1A1*–*PDGFB* fusion uterine sarcomas was older, ranging from 43 to 82 years (average at 56.7 years, median at 53.5 years). IHC features usually provided more clues for differential diagnosis, as CD34 was usually stained remarkably positive, while TRK, S100, myogenic markers, and hormone receptors were often stained negative. Although in most cases IHC staining is a simple and cost-effective method to assist diagnosis, confirmatory FISH or gene sequencing is mandatory in cases that are hard to identify. Due to the rarity of this tumor, limited experience with clinical treatment and prognosis, and lack of knowledge about the effectiveness of targeted therapy, it was particularly critical to correctly identify the tumor as the first step.

Under a microscope, a relatively sparse area of tumor cells with rare mitoses inspected could also be confused with leiomyoma. However, IHC markers for smooth muscle differentiation (desmin, caldesmon, and SMA) stained negative could exclude benign leiomyoma. LMS usually displayed moderate-to-severe nuclear heteromorphism, active mitoses, and remarkable necrosis and stained positive for the markers of smooth muscle differentiation (23), which were inconsistent with *COL1A1*–*PDGFB* fusion uterine sarcomas. The tumor cells of HGESS were often smaller with irregular or tongue-like invasion into the myometrium, and CCND1 and BCOR were often positive on IHC. They usually presented with specific gene fusion of *YWHAE*–*NUTM2 A/B* fusion and *ZC3H7B*–*BCOR* fusion. Other mutations such as *EPC1*, *SUZ12*, *BRD8*, *PHF1*, *TPR*, *LMNA*, *TPM3*, *RBPMS*, *EML4*, and *STRN* were also reported (30–32).

The prognosis for *COL1A1*–*PDGFB* fusion uterine sarcomas does not seem optimistic so far, as two patients of the five cases reported before have died. Both of them were at advanced clinical stages IIIB and IVA. Our patient (case 6 in **Table 2**) did not show any recurrence or progression for the past 6 months. The rarity of *COL1A1*–*PDGFB* fusion uterine sarcomas occurring in the female genital tract and unspecific morphology, especially without molecular tests, resulted in frequent misdiagnosis. Misdiagnosis could be one of the main reasons for poor prognosis, as adequate adjuvant therapy was delayed or missed for these patients. Therefore, awareness is encouraged when morphology and IHC markers do not match, and assistance from molecular tests (especially RNA sequencing for gene fusion in sarcoma) is critical for precise diagnosis.

As the clinical signs and symptoms in patients with *COL1A1*–*PDGFB* fusion uterine sarcomas in the female genital tract were usually silent in early stages, two out of six patients were initially diagnosed at late stages (IIIB and IVA in cases 2 and 4) as shown in **Table 2**. These two patients died of the disease at 60 and 34 months on follow-up. In case 4, the lack of response to chemotherapy prompted genomic testing for potential targeted therapies. It was at that time the *COL1A1*–*PDGFB* fusion was identified. Treatment with imatinib was initiated and continued for 6 months. The effects lasted and achieved the peak at the 11-month follow-up, as the intrabdominal mass reduced in size from 22.4 to 6.5 cm. CT progression was noticed at the 14-month follow-up after initiation of imatinib, as multiple abdominal masses that previously decreased in size grew back rapidly. Further investigations of more targeted therapy at *COL1A1*–*PDGFB* fusion are urgently needed to improve prognosis. Routine physical examination and clinical identification with the coordination of gynecologists and radiologists are crucial to guarantee early diagnosis and prompt treatment.

As for the surgical approach, the risk of inadvertent dissemination of occult malignancies of presumed benign tissue must be considered, as *COL1A1*–*PDGFB* fusion uterine sarcomas could be judged as benign leiomyoma on imaging (33). Morcellation should be avoided or carefully performed under the protection of a specimen bag. Laparotomy, colpotomy, or laparoscopic hysterectomy with contained specimen extraction through the vagina is appropriate.

This study expands the clinicopathological features of *COL1A1*–*PDGFB* fusion uterine sarcomas of the cervix, adding the first Chinese case to the five reported cases and highlighting a potential pitfall in the morphological differential diagnosis with *NTRK* fusion uterine sarcoma, leiomyoma, LMS, HGESS, and undifferentiated uterine sarcoma. A lack of knowledge has been seldom discussed previously. The prognosis for *COL1A1*–*PDGFB* fusion uterine sarcomas does not seem optimistic so far, as the current clinical evidence, long-term follow-up of these patients, and more clinical analyses with bigger sample size are urgently needed to better study the prognosis of this particularly rare type of uterine sarcoma. More investigations are warranted to clarify the pathogenesis and development of this disease and help improve the prognosis.

## Conclusion

The new categorization of uterine spindle cell sarcomas in 2009 started a new era of pathological diagnosis depending on molecular features from classical morphology. With only five cases previously reported, we presented the sixth case of *COL1A1*–*PDGFB* fusion uterine sarcoma in the female genital tract. Its pathological morphology is easily confused with benign uterine leiomyoma. Moreover, other mesenchymal malignancies such as *NTRK* fusion uterine sarcoma, LMS, and HGESS also need to be differentiated. The assistance by immunohistochemistry and molecular detection is critical for precise pathological diagnosis of different categories of uterine sarcoma. Precise identification could allow patients to benefit from further treatment, especially targeted therapy such as imatinib.

## Data availability statement

The original contributions presented in the study are included in the article/**Supplementary Material**. Further inquiries can be directed to the corresponding authors.

## Ethics statement

Written informed consent was obtained for the publication of this case report.

## Author contributions

Author Contributions: Conceptualization, YN and YS. Methodology, YN and LL. Software, LL. Clinical resources, YN and LL. Lab work, SW, FZ and LL. Writing—original draft preparation, YS and LL. Writing—review and editing, YN and YS. Visualization, HS and FM. Supervision, YN and YS. All authors contributed to the article and approved the submitted version.

## Supplementary material

Supplementary information of genes covered by the sarcoma gene panel
AKT2, AKT3, ALK, AR, AXL, BCL2, BCOR, BCR, BRAF, BRCA1, BRCA2, BRD4, CD74, CDK12, CDKN2A, CIC, CTNNB1, DDR2, EGFR, ERBB2, ERBB4, ERG, ESR1, ETV1, ETV6, FGFR1, FGFR2, FGFR3, FGR, FLT3, FOXO1, GLI1, JAK2, JAK3, KIT, KMT2A, KRAS, MDM4, MET, MSH2, MYC, NF1, NOTCH1, NOTCH2, NRG1, NTRK1, NTRK2, NTRK3, PDGFB, PDGFRA, PDGFRB, PIK3CA, PPARG, PTEN, RAD51B, RAF1, RARA, RB1, RET, ROS1, SMARCB1, STK11, TERT, TFE3, TMPRSS2, WT1, ETV4, ETV5, EWSR1, MYB, NUTM1, EZR, SLC34A2, SDC4, ACTB, ATF1, CAMTA1, COL1A1, COL1A2, CREB3L1, CREB3L2, CSF1, DNAJB1, FEV, FLI1, FUS, JAZF1, LPP, MGEA5, NAB2, NCOA2, NOTCH4, NR4A3, PHF1, PLAG1, PRKACA, RANBP2, RELA, RIPK4, RPS6KB2, SS18, SSX1, USP6, YWHAE, HMGA2, TAF15, TFG, CCNB3, EPC1, MEAF6, MKL2, STAT6, TCF12, NUP214, PRKACB, PRKCA, TACC3, DDIT3, SLC45A3, NCOA1, EIF3E, RSP02, PAX8, MAML2, PAX3, PAX7, PTPRK, RSPO3, NUP107, PBX1, C110RF95, COL6A3, MAML3, MYBL1, PLK2, PRDM10, PRKCB, TFEB, TRIO, VGLL2, GRB7, MAST1, MAST2, BRD3, CDH11, ESRP1, MYH9, YAP1.

Supplementary Figure 1 Pelvic MRI showed that an oval mass protruding from the cervix to the vagina and compressed on the anterior bladder with a slightly higher signal **(A)**. The mass was significantly enhanced, remarkably higher than that of the uterus post-enhancement **(B)**.

Supplementary Figure 2 On gross examination of the specimen, the tumor was mainly located on anterior lip of the cervix, growing in a pushing manner toward the vagina. The cross-section of the mass was firm and white-whorled with relatively clear boundary.
