# Supplementary figures and images for: Case Report: A case of COL1A1–PDGFB fusion uterine sarcoma at cervix and insights into the clinical management of rare uterine sarcoma
Source: Front Oncol. 2023 Mar 13;13:1108586. doi: 10.3389/fonc.2023.1108586 (PMC10042132; doi:10.3389/fonc.2023.1108586)

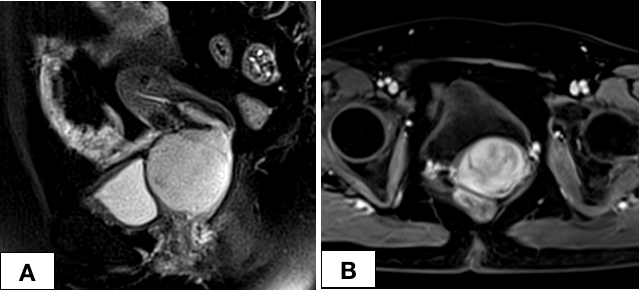

Supplement: Supplementary information of genes covered by the sarcoma gene panel — AKT2, AKT3, ALK, AR, AXL, BCL2, BCOR, BCR, BRAF, BRCA1, BRCA2, BRD4, CD74, CDK12, CDKN2A, CIC, CTNNB1, DDR2, EGFR, ERBB2, ERBB4, ERG, ESR1, ETV1, ETV6, FGFR1, FGFR2, FGFR3, FGR, FLT3, FOXO1, GLI1, JAK2, JAK3, KIT, KMT2A, KRAS, MDM4, MET, MSH2, MYC, NF1, NOTCH1, NOTCH2, NRG1, NTRK1, NTRK2, NTRK3, PDGFB, PDGFRA, PDGFRB, PIK3CA, PPARG, PTEN, RAD51B, RAF1, RARA, RB1, RET, ROS1, SMARCB1, STK11, TERT, TFE3, TMPRSS2, WT1, ETV4, ETV5, EWSR1, MYB, NUTM1, EZR, SLC34A2, SDC4, ACTB, ATF1, CAMTA1, COL1A1, COL1A2, CREB3L1, CREB3L2, CSF1, DNAJB1, FEV, FLI1, FUS, JAZF1, LPP, MGEA5, NAB2, NCOA2, NOTCH4, NR4A3, PHF1, PLAG1, PRKACA, RANBP2, RELA, RIPK4, RPS6KB2, SS18, SSX1, USP6, YWHAE, HMGA2, TAF15, TFG, CCNB3, EPC1, MEAF6, MKL2, STAT6, TCF12, NUP214, PRKACB, PRKCA, TACC3, DDIT3, SLC45A3, NCOA1, EIF3E, RSP02, PAX8, MAML2, PAX3, PAX7, PTPRK, RSPO3, NUP107, PBX1, C110RF95, COL6A3, MAML3, MYBL1, PLK2, PRDM10, PRKCB, TFEB, TRIO, VGLL2, GRB7, MAST1, MAST2, BRD3, CDH11, ESRP1, MYH9, YAP1. [file Image_1.tif]
